# Multimodal MRI for early diabetic mild cognitive impairment: study protocol of a prospective diagnostic trial

**DOI:** 10.1186/s12880-016-0152-x

**Published:** 2016-08-24

**Authors:** Ying Yu, Qian Sun, Lin-Feng Yan, Yu-Chuan Hu, Hai-Yan Nan, Yang Yang, Zhi-Cheng Liu, Wen Wang, Guang-Bin Cui

**Affiliations:** Department of Radiology, Tangdu Hospital, Fourth Military Medical University, 569 Xinsi Road, Xi’an, 710038 China

**Keywords:** Type 2 diabetes mellitus, Mild cognitive impairment, Neuroimaging techniques, Prodromal diabetic stage, Microstructural alterations, Microvascular alterations

## Abstract

**Background:**

Type 2 diabetes mellitus (T2DM) is a risk factor for dementia. Mild cognitive impairment (MCI), an intermediary state between normal cognition and dementia, often occurs during the prodromal diabetic stage, making early diagnosis and intervention of MCI very important. Latest neuroimaging techniques revealed some underlying microstructure alterations for diabetic MCI, from certain aspects. But there still lacks an integrated multimodal MRI system to detect early neuroimaging changes in diabetic MCI patients. Thus, we intended to conduct a diagnostic trial using multimodal MRI techniques to detect early diabetic MCI that is determined by the Montreal Cognitive Assessment (MoCA).

**Methods:**

In this study, healthy controls, prodromal diabetes and diabetes subjects (53 subjects/group) aged 40-60 years will be recruited from the physical examination center of Tangdu Hospital. The neuroimaging and psychometric measurements will be repeated at a 0.5 year-interval for 2.5 years’ follow-up. The primary outcome measures are 1) Microstructural and functional alterations revealed with multimodal MRI scans including structure magnetic resonance imaging (sMRI), resting state functional magnetic resonance imaging (rs-fMRI), diffusion kurtosis imaging (DKI), and three-dimensional pseudo-continuous arterial spin labeling (3D-pCASL); 2) Cognition evaluation with MoCA. The second outcome measures are obesity, metabolic characteristics, lifestyle and quality of life.

**Discussion:**

The study will provide evidence for the potential use of multimodal MRI techniques with psychometric evaluation in diagnosing MCI at prodromal diabetic stage so as to help decision making in early intervention and improve the prognosis of T2DM.

**Trial registration:**

This study has been registered to ClinicalTrials.gov (NCT02420470) on April 2, 2015 and published on July 29, 2015.

## Background

The prevalence of type 2 diabetes mellitus (T2DM) is rapidly increasing, making T2DM currently a major health challenge all over the world. Epidemiological studies showed that the T2DM was associated with a 1.5–2.5-fold increased risk of dementia [1]. Mild cognitive impairment (MCI) frequently occurs in T2DM population at a rate of 17.2 % [2] and consequently leads to dementia [3–5]. Moreover, MCI occurs at as early as the prodromal diabetic stage, making the early diagnosis of MCI an important strategy for decision making in T2DM treatment, especially for the prevention of T2DM related dementia.

MCI is currently diagnosed based on clinical criteria [6], mainly the cognition evaluation, however, with bias derived from the subjective nature of these cognition assessing tools. Other relatively objective neuroimaging biomarkers are proposed to detect T2DM related brain alterations that are supposed to be the underlying mechanisms for MCI or dementia. T2DM related MCI may be partly due to neuroanatomical alterations revealed by structural magnetic resonance imaging (sMRI), including atrophy in prefrontal cortex and the anterior cingulate [7–9]. Findings from diffusion tensor imaging (DTI) studies further indicated decreased fractional anisotropy values in frontal white matter [10], cingulum bundle, uncinate fasciculus [11], and anterior limb of internal capsule [12] in T2DM patients. Resting-state functional magnetic resonance imaging (rs-fMRI) studies demonstrated disrupted functional connectivity within the default mode network and decreased spontaneous brain activity in the occipital lobe and post central gyrus in T2DM patients [11, 13, 14]. A most recent study suggested the impaired macromolecular protein pools in fronto-striato-thalamic circuits in T2DM [15]. However, these findings can only be detected at the late stage of T2DM, thus cannot be used to screen MCI at the prodromal diabetic stage.

Previous studies suggested that the brain microvascular alterations might explain MCI. A positron emission tomography-computed tomography study revealed that the focal brain blood decrease occured at as early as several years before Alzheimer`s disease diagnosis and was accompanied with the MCI progression [16]. On the contrary, a trend to a slight cerebral blood flow (CBF) increase in bilateral hippocampi and the posterior cingulate gyrus among patients with MCI was noticed using dynamic susceptibility contrast MRI [17]. The major shortcoming of these methods to measure CBF is the use of radio-labeled contrast agents, thus, not suitable for screening brain microvascular alterations in the prodromal diabetic population. Three-dimensional pseudo-continuous arterial spin labeling (3D-pCASL), a non-invasive and non-contrast perfusion imaging method [18], is commonly used to measure CBF alterations and demonstrates equivalent diagnostic performance to Single-photon emission computed tomography (SPECT) for Alzheimer`s disease [19].

Thus, we designed the current prospective diagnostic trial for 2 purposes. First, to investigate the structure, functional alterations and gray/white matter integrity with sMRI, fMRI and diffusional kurtosis imaging (DKI, also measures non-Gaussian diffusion and may provide additional and complementary information to DTI [20]) in prodromal diabetic population, respectively. Second, to investigate the early brain microvascular alterations by using 3D-pCASL.We hope to establish an integrated diagnosing system for early MCI in prodromal diabetic population by using multimodal MRI techniques.

## Methods/Design

**Fig. 1:**
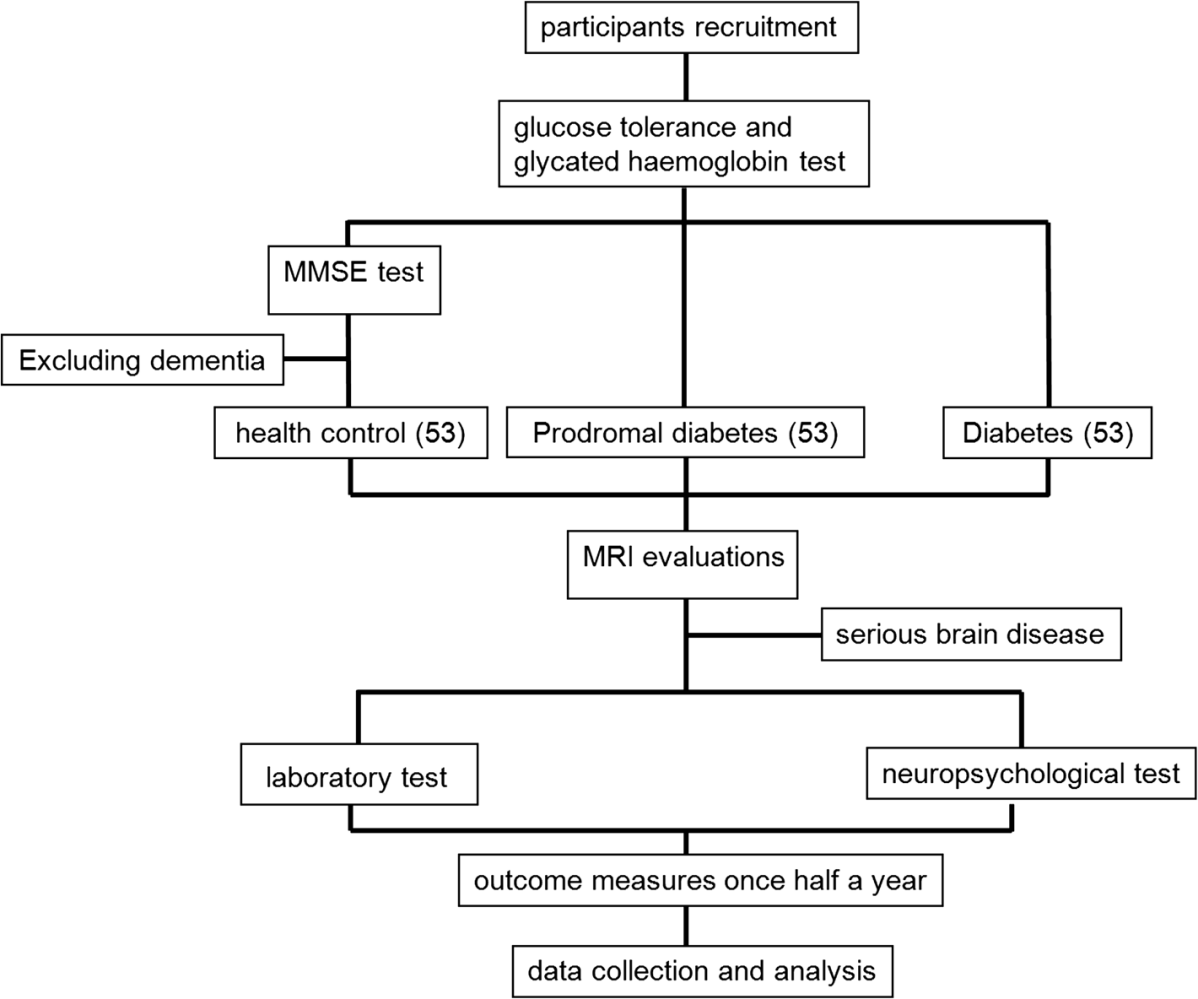
Flowchart of the current prospective diagnostic trial

The scheme of the current prospective trial is described in Fig. 1 and the following part.

### Subject

#### Subjects inclusion

Participants with a minimum of the senior high school education will be prospectively recruited in one of the three groups: healthy control (HC), prodromal diabetes, or T2DM group according to the following inclusion criteria. *HC group*: Individuals with normal HbA1c and blood glucose level and no evidence of T2DM will be recruited into this group. Their fasting plasma glucose (FPG) levels are < 5.6 mmol/L, and 2 h fasting glucose (PG) levels for oral glucose tolerance test (OGTT) are < 7.8 mmol/L. *Prodromal diabetes group*: Individuals who have the impaired fasting glucose (IFG, FPG 5.6-6.9 mmol/L) or impaired glucose tolerance (IGT, 2 h PG for OGTT 7.8-11.1 mmol/L, but not meet the diagnostic criteria for T2DM will be recruited in this group. *T2DM group*: Individuals will be recruited into this group if they fall in any one of the following criteria: (1) FPG ≥ 7.0 mmol/L or, (2) 2 h PG for OGTT ≥11.1 mmol/L or, (3) random PG ≥11.1 mmol/L in the presence of typical diabetes symptoms.

#### Subjects exclusion

Subjects will be excluded because of any one of the following conditions. 1) dementia, which is defined by the <21 score in mini-mental state examination (MMSE); 2) obese, which is defined by the > 28 score of the body mass index; 3) other type of diabetes, such as type 1 diabetes mellitus and secondary diabetes mellitus; 4) serious brain diseases, such as significant head trauma, tumor, meningitis or central nervous system inflammatory lesions and vascular complications (e.g., infarction and encephalomalacia foci) clues which may be detected with conventional MR scan; 5) MRI contraindications; or 6) taking psychoactive or steroid hormones drugs during the past 3 months.

Participants will receive laboratory, neuropsychological tests as well as multimodal brain MRI scans during a comprehensive 6 h evaluation completed over three visits within 2 weeks.

### Neuropsychological assessments

All individuals received the following a battery of neuropsychological tests within 2 weeks before imaging: MMSE, Montreal Cognitive Assessment (MoCA), Frontal Assessment Battery (FAB), Hamilton Anxiety Scale (HAS), Self-Rating Depression Scale (SDS), the World Health Organization Quality of Life assessment (WHOQOL).

### Magnetic resonance brain imaging

#### Imaging protocol

Imaging data will be acquired using a 3.0-T MRI system (MR750; GE Healthcare, Milwaukee, WI, USA) using an 8-channel head coil array with foam padding to restrict head motion. For each participant, conventional brain T1-weighted (T1WI), T2-weighted (T2WI) and fluid-attenuated inversion recovery (FLAIR) images will be obtained to exclude serious brain diseases. All of the sequences we mentioned use the isotropic spatial resolution. All MR images will be assessed by two experienced radiologists (with over 5-year experience).

#### sMRI

Following the pilot scan, for brain structure segmentation and image registration to a brain anatomy template, an axial 3D brain volume imaging (3D-BRAVO) will be acquired for T1WI, according to the following parameters: echo time (TE) = 3.2 ms, repetition time (TR) = 8.2 ms, inversion time(TI) = 450 ms, flip angle (FA) = 12°, field of view (FOV) =256 × 256 mm^2^, matrix = 256 × 256, slice thickness = 1 mm, slice number = 188. The whole procedure will cost 4 min 10 s.

#### 3D-pCASL

According to the White Paper of the International Society for Magnetic Resonance in Medicine (ISMRM) perfusion study group [21], Perfusion images will be obtained using a 3D-pCASL technique [22] with the following parameters: 512 sampling points on eight spiral arms, TR = 4886 ms (post label delay (PLD) = 2025 ms), TE = 10.5 ms, slice number = 40, FOV = 256 × 256 mm^2^, matrix = 128 × 128, slice thickness = 2.0 mm, number of excitations (NEX) = 3, FA = 111°. The bottom of the slab should be positioned at the bottom of the cerebellum, with coverage of the entire cerebrum. This sequence is pseudo-continuous labeling, background suppression, and a segmented three-dimensional readout without vascular crushing gradients. A simplified model will be used to calculate and present both label/control difference images and cerebral blood flow in absolute units. The whole scan will cost 4 min.

#### DKI

DKI datasets will be acquired using a spin-echo single-shot diffusion tensor echo planar imaging (SE-SS-DT-EPI) sequence with a clinically oriented protocol. The acquisition parameters will include three b-values of 0, 1000, and 2000 s/mm^2^, 10 b_0_ images, 25 diffusion gradient directions, TR/TE = 5800/77 ms, FOV =256 × 256 mm^2^, matrix = 128 × 128, slice thickness = 2 mm, FA = 90°, slice number = 48, in-plane spatial resolution = 2 × 2 mm^2^. The acquisition time for this protocol will be 5 min 54 s.

#### rs-fMRI

rs-fMRI images will be collected using an EPI sequence with the following parameters: TR = 2000 ms, TE = 30 ms, FA = 90°, slice number = 33, inter slice gaps = 0 mm, FOV = 192 × 192 mm^2^, matrix = 64 × 64, slice thickness = 3 mm, in-plane spatial resolution = 3 × 3 mm^2^. Participants will be asked to lie quietly in the scanner with their eyes closed and thinking nothing during data acquisition. The scan will last for 6 min 10 s.

### MRI image analysis

#### sMRI data analysis

Measurements of total intracranial, whole brain and ventricular volumes will be calculated using volumetric output measures and semi-automated segmentation from FreeSurfer ASEG analysis [23]. Whole brain and ventricular volumes will be normalized to the whole intracranial volume to adjust for variability due to head size. The normalized volumes were used for among group comparisons to evaluate the significant group differences.

#### 3D-pCASL data analysis

Data will be preprocessed using Statistical parametric mapping (SPM12) (http://www.fil.ion.ucl.ac.uk/spm/software/spm12/), in which 3D-BRAVO and 3D-pCASL images will be corrected for gradient nonlinearities in all directions. Realignment, coregistration and segmentation will be included in the preprocessing. ASL images will be registered to the brain extracted from the 3D-BRAVO. Mean whole-brain CBF values will be calculated in the mask, converted to quantitative CBF maps with the unit of mL/100 g/min. And then it will be normalized to Montreal Neurological Institute (MNI) space with a 3-mm isotropic resolution, and smooth with an isotropic kernel of 8 mm. Multiple comparisons correction will be used (*P* < 0.05) to investigate voxel-wise CBF differences among groups with a minimum size of 50 pixels. All results will be expressed in the MNI space. Mean regional CBF values will be measured in each interested gyri.

#### DKI data analysis

Data will be processed using a combination of in-house image processing tools developed in MATrix LABoratory(MATLAB) (Mathworks, Natick, USA) and tools available as part of Freesurfer (http://surfer.nmr.mgh.harvard.edu) and FMRIB’s Software Library (FSL FSL version 5.0, http://www.fmrib.ox.ac.uk/fsl). The diffusion dataset will be corrected to get potential 3D head motion and eddy current distortion using FSL eddy correct. The toolbox implemented in MATLAB will be used to deal with diffusional kurtosis tensors [24]. Coefficients from these tensors will be estimated for each voxel using a previously described homogenous polynomials approach, in which positive diffusivity function, positive-definite estimated diffusion tensor, and constrained estimated apparent kurtosis will be done [24, 25].

#### rs-fMRI

rs-MRI imaging data will be preprocessed in the toolbox of MATLAB R2012b Data Processing Assistant for Resting-State fMRI (DPARSF, http://www.restfmri.net/forum/DPARSF), SPM12 and rs-fMRI data analysis toolkit (REST1.8; http://www.restfmri.net) will be selected to deal with the images. Slice timing and realignment for head motion correction will be performed. Any images with head motion 3.0-mm translation or 3.0° rotation in any direction will be excluded. Amplitude of low frequency fluctuation (ALFF) and regional homogeneity (ReHo) images will be estimated in the REST software as described in previous studies [26, 27]. For ALFF and ReHo analysis, the resampled images will be smoothed, linear trend and band-pass filtering (0.01–0.08 Hz). All the results will be expressed in the MNI space, and the data can be read and described through the REST software.

Functional connectivity among groups is also from the rs-MRI imaging data. Data analysis will be carried out using MELODIC of FSL. Images will firstly be slice-time and motion corrected followed by normalization and spatial smoothing. The number of components is fixed at 20 [28]. The group ICA was repeated several times using unique randomly resampled data to ensure stability of 20 independent components. A meta-ICA analysis will be then carried out using all iterations of the group ICA components to extract the 20 spatially independent components consistently identified across the group ICA runs. Dual regression will be applied to each individual’s preprocessed datasets using the 20 group components. The first regression model uses the template as a spatial predictor for the participant’s 4D data. The second regression equation estimated the individual regression weights in the spatial domain. Individual default mode network component maps will be selected to carry out group-level statistics.

### Follow up

A follow-up assessment will be performed within three years after individual’s first visit with changes of imaging presentations and other outcomes in the three groups as time goes on (up to 36 months) be compared. Individuals will come back to the medical center once half a year for countercheck. All the protocols and evaluations are the same with that they get at the first time.

### Sample size calculation and statistics

Power calculations have been estimated with reference to other studies that used similar end points [21, 29], according to the formula for estimating the variances of mean values from multiple groups [29]. A sample size of 40 patients per group will be suitable with a power of 0.8, an alpha significance level of 0.05 (two-side). In our group, longitudinal changes of this effect size have been shown previously in groups larger than 10 subjects. Given a drop-out ratio of included subjects, at 25 %, into account, the total sample size is 53 in each group.

Demographic clinical, laboratory, cognitive, and neuropsychological data will be analyzed using SPSS 22.0 (SPSS, Inc.). One-way analysis of variance or *χ*^2^ test will be used to explore differences of these data among three groups (HC, prodromal diabetes and diabetes). Neuropsychological difference among them will be performed with analysis of covariance (ANCOVA) using demographic factors (gender, age, and years of education) as covariates. *Post-hoc* comparisons will then be performed with all demographic factors (gender, age, and years of education) as covariates. *P* < 0.05 is considered as significant. It is reported ReHo and ALFF values are affected by the structure alterations (e.g., atrophy) [30]. We will take ventricular volume as a covariance to rule out such influence.

For images analysis, one-sample t tests will be performed on the individual corresponding maps for each group using SPM12 to explore the differences within groups. Dunnett-*t* test will be performed for differences among case groups and healthy control group. The statistical threshold of the two test will both be set at *P* <0.005 which corresponded to a corrected *P* <0.005 (multiple comparisons with family-wise error).

Linear regression analysis is performed across the T2DM groups and prediabetes groups to assess relationships between imaging values and cognitive testing scores. When the most sensitive parameters are found, a receiver operating characteristic (ROC) curve will be generated to achieve the sensitivity and specificity of the certain parameter for early detection of the cognitive decline.

### Primary outcome measure

1. Network connectivity among multiple brain regions and neuroimaging biomarkers with the 2.5 years’ follow-up at 0.5 year interval. [Time Frame: subjects will be assessed six times (once half a year, up to 2.5 years)]
2. Differences in macro-structural and micro-structural network connectivity among healthy controls, prodromal diabetes and diabetes will be evaluated. These MRI measures include volumetric characteristics (e.g., hyper-intensities, white matter lesions, atrophy), quantitative measures (e.g., T2 relaxation times, mean diffusivity, fractional anisotropy, mean kurtosis), functional characteristics (e.g., activated regions, cerebral blood flow), network properties (e.g., functional and structural connectivity, graph-theoretical measures). [Time Frame: subjects will be assessed six times (once half a year, up to 2.5 years)]

### Secondary outcome measures

1. *Evaluation of obesity and the changes in the state of obesity at 2.5 years*. Simple evaluation of obesity will include: body mass index will be calculated as the weight (kg) divided by the square of the height (m^2^). Waist circumference will be taken as the minimum circumference between the umbilicus and xiphoid process and measured to the nearest 0.5 cm. [Time Frame: subjects will be assessed six times (once half a year, up to 2.5 years)]
2. *Metabolic characteristics and their changes at 2.5 years*. Simple metabolic characteristics will include: oral glucose tolerance test, C peptide releasing test and insulin releasing test, homeostasis model assessment of insulin resistance,insulin secretion of homeostasis model assessment and homeostasis model assessment-β. What’s more, cardiovascular risk factors (e.g., albumin, creatinine, total cholesterol, low density lipoprotein (LDL)- and high density lipoprotein (HDL)-cholesterol, triglycerides, glycosylated hemoglobin) will be assessed. [Time Frame: subjects will be assessed six times (once half a year, up to 2.5 years)]
3. *Mental health and the changes in the scores of the scales at 2.5 years*. To evaluate the mental health, a series of psychiatric evaluation scale (e.g., MMSE, MoCA,HAS, SDS, WHOQOL) will be assessed. [Time Frame: subjects will be assessed six times (once half a year, up to 2.5 years)]
4. *Lifestyle and its changes at 2.5 years*. Lifestyle specifics, including alcohol consumption, smoking behavior and mobility and exercise habit will be obtained. At the same time, quality of life will be obtained through a questionnaire. [Time Frame: subjects will be assessed six times (once half a year, up to 2.5 years)]

## Discussion

The current study evaluates various characteristics of diabetes, especially the central nervous system image presentations in different stage of diabetes progression, and aims to establish an imaging evaluation system for detecting and evaluate MCI in diabetes. Benefits and limitations of this study are discussed below.

It is reported by most researchers that diabetes result in regional hypoperfusion. However, Henry Rusinek and his colleagues found that no hypoperfusion exists in diabetes group, in contrast to hypoperfusion in insulin resistant but not diabetes group [29]. Since insulin resistant is one of main mechanisms of diabetes, we are still uncertain why the perfusion data differs between the two groups. The current study will follow up individual’s characteristics in various areas, especially the image presentation for not less than 3 years. Natural progression of diabetes is monitored. Reperfusion or compensation result in diabetes will be explored. We may discovery the unrevealed mechanism for the divergent between IR and diabetes group.

Structure changes are common in advanced T2DM. It may also exist at prediabetes stage. The volume changes should be used to be one of the covariant to prevent its effect to signal intensity. In current study, we take cerebral ventricle volume, which may represent the structure changes of the whole brain, as a covariant. DKI reveals the non-Gaussian characteristics of gray and white matter [31]. Imaging reports of this respect in diabetes are limited. We undergo the examination of DKI, instead of DTI to explore the real world of microstructure.

Various imaging researches of diabetes have been reported. Most of them are studies of one or two protocols. In our study, multi-modal sequences will be conducted and analyzed at the same time. It may be revealed which one or combined protocols may express the highest area under curve by drawing ROC curve. Corresponding sensitivity and specificity are calculated, and then we will get the cutoff values to diagnose MCI.

MoCA is verified to be a valid scale for MCI in diabetes in China [32]. Different dimensions scores of MoCA will be calculated and analyzed together with imaging presentations. Therefore, we may detect the functional node for different cognitive dimensions. The association may help to speculate prognosis. The neuropsychological test battery comprises another four scales. They test the condition of cognition, affection and life style. Different cognitive-related circuits overlap and interact significantly. Therefore, affective and life style state evaluation is necessary for matching the changes of cerebral functional imaging presentations.

Treatment strategies vary significantly. According to our preliminary experiment, patients took metformin either alone or in combination with other drugs. Some of the diabetes went on insulin treatment. Part of the prediabetes group took statin-related drugs. Antihypertensive drugs were also taken by some individuals to control their blood pressure. Medications related to cardiovascular disease affect cerebral perfusion significantly [27], and they may play a role in neuronal plasticity as well [26]. In the current study, we have not taken medication therapies as covariant for some practice and ethics issues. The reliability of results may be affected by the disaccord of medication therapies.

Future studies should focus on comparing the imaging characteristics between MCI converted group and no converted group. Gene type may serve as a control condition. Other techniques such as using MRS for the metabolic data could be useful and should be evaluated as well. The consistency of therapeutic regimen should also be guaranteed.

## Abbreviations

ALFF: Amplitude of low frequency fluctuation
CBF: Cerebral blood flow
3D-BRAVO: Three dimensional brain volume imaging
DKI: Diffusional kurtosis imaging
3D-pCASL: Three-dimensional pseudo-continuous arterial spin labeling
DTI: Diffusion tensor imaging
FAB: Frontal assessment battery
FLAIR: Fluid-attenuated inversion recovery
FPG: Fasting plasma glucose
HAS: Hamilton anxiety scale
HC: Healthy control
IFG: Impaired fasting glucose
IGT: Impaired glucose tolerance
MCI: Mild cognitive impairment
MMSE: Mini-mental state examination score
MNI: Montreal Neurological Institute
MoCA: Montreal cognitive assessment
OGTT: Oral glucose tolerance test
ReHo: Regional homogeneity
ROC: Receiver operating characteristic
rs-fMRI: resting-state functional magnetic resonance imaging
SDS: Self-rating depression scale
sMRI: structure magnetic resonance imaging
SPECT: Single-photon emission computed tomography
SPM12: Statistical parametric mapping 12
T2DM: Type 2 diabetes mellitus
T1WI: T1-weighted imaging
T2WI: T2-weighted imaging
WHOQOL: The World Health Organization quality of life assessment
